# Implementation of occupational safety and health services and telehealth in Peru: a narrative review

**DOI:** 10.47626/1679-4435-2025-1405

**Published:** 2025-07-08

**Authors:** Jonh Astete-Cornejo, Miguel Angel Burgos-Flores, Liliana Cruz-Ausejo, Jimmy Andreyvan Cainamarks-Alejandro, Juan Israel Ambrosio-Melgarejo, Jaime Rosales-Rimache

**Affiliations:** 1 Centro Nacional de Salud Ocupacional y Protección del Ambiente para la Salud, Subdirección de Medicina del Trabajo y Ambiental, Instituto Nacional de Salud, Lima, Peru

**Keywords:** occupational health, occupational safety, occupational health services, telemedicine, mHealth., saúde ocupacional, segurança ocupacional, serviços de saúde do trabalhador, telemedicina, mSaúde.

## Abstract

Although occupational health aims to protect the well-being of workers, challenges persist in workplaces, such as the provision of occupational safety and health services and the availability of occupational telehealth. This study’s objective was to examine the implementation of occupational safety and health services and telehealth in Peru. A review was conducted of technical documents and regulations related to occupational safety and health services and occupational telehealth applicable to the Peruvian context. Forty percent of Peruvian workplaces lack occupational safety and health services, reflecting a similar issue across Latin America and Europe. Barriers such as corporate commitment and financing were identified. Occupational telehealth was utilized during the COVID-19 pandemic, highlighting its potential to strengthen occupational safety and health services. The lack of occupational safety and health services and occupational telehealth can be overcome through regulatory support, enforcement, and incentives for employers.

## Abstract (pt)

A saúde ocupacional visa a proteger o bem-estar dos trabalhadores; no entanto, persistem desafios nos locais de trabalho, como a oferta de serviços de segurança e saúde ocupacional e telessaúde ocupacional. O estudo pretendeu examinar a implementação de serviços de segurança e saúde ocupacional e telessaúde ocupacional no Peru. Realizou-se uma revisão de documentos técnicos e regulamentos relacionados a serviços de segurança e saúde ocupacional e telessaúde ocupacional aplicáveis ao contexto peruano. O Peru tem 40% dos locais de trabalho sem serviços de segurança e saúde ocupacional, o que reflete um problema semelhante na América Latina e na Europa. Identificaram-se barreiras como o compromisso empresarial e o financiamento. Durante a pandemia de covid-19, utilizou-se telessaúde ocupacional, destacando seu potencial para fortalecer os serviços de segurança e saúde ocupacional. Existe uma lacuna na implementação de serviços de segurança e saúde ocupacional e telessaúde ocupacional, que pode ser superada por meio de suporte regulatório, fiscalização e incentivos aos empregadores.

## INTRODUCTION

Occupational health is the discipline of ensuring the physical, mental, and social well-being of workers in their workplaces.^1^ According to the International Labour Organization (ILO), nearly 3 million deaths occur annually due to work-related accidents or illnesses, in addition to over 390 million non-fatal work-related injuries, resulting in an increasing disease burden among the working population, along with economic loss and added strain on health care systems.^2,3^ It is crucial to improve working conditions to prevent these risks and promote a culture of workplace safety. Scientific research in this field provides valuable insights for designing health policies and addressing challenges that affect both productivity and safety in the workplace. However, further evidence is needed in this field, particularly in regions like South America, where there is lower development in terms of preventive technologies or occupational health intervention, including telehealth.^2,4^

Occupational safety and health services (OSHS) are functional units through which employers implement measures to prevent and control occupational hazards. A company may establish these services internally or externally and they are staffed by occupational health and safety professionals.^5^ Although directly dependent on the employer, their scope and composition are regulated by the government. It is worth noting that occupational health professionals within OSHS require additional training and experience in occupational health and safety, which may include technical, professional, or specialized education, in addition to their health care background.^5,6^ Despite the evident importance of having OSHS in every workplace, especially those with potentially fatal occupational hazards, global coverage remains inadequate. Even in regions with early implementation of occupational health regulations, such as the European Union, appropriate coverage is as low as reach 20% of the working population in some countries.^5^

In Peru, Law No. 29783 established a regulatory framework for the development of occupational health on a national level, as well as the functions and responsibilities of the agents within the occupational health and safety system, including OSHS.^7^ Before this law was passed, only high-risk sectors such as mining had medical services and hospitals for their workforce. However, this regulation’s enactment has led to workplaces establishing their own OSHS, with up to 80% compliance in large companies whose activities involve higher risk.^8^ During the pandemic, and given the deficient implementation of OSHS in the majority of companies and public entities, the Peruvian government, through the National Center for Occupational Health and Environmental Protection for Health (Centro Nacional de Salud Ocupacional y Protección del Ambiente para la Salud-Instituto Nacional de Salud [CENSOPAS-INS]), issued “Guidelines for the surveillance, prevention, and control of worker health regarding COVID-19 exposure”.^9^ These guidelines mandated the hiring of health care professionals according to the size of the company if it did not already provide its own OSHS during the health emergency. Despite this measure, which relaxed the requirements for occupational health professionals due to the pandemic, between 20% and 40% of businesses had not hired the minimum number of professionals or did not have an OSHS, especially in high-risk sectors such as health care and mining.^10-12^

In contrast, telehealth had a positive impact as a health care strategy during the pandemic, experiencing a sixty-fold increase in demand, including the initiation of telehealth services focused on mental health.^13^ These telehealth services were mainly implemented by the Ministry of Health (Ministerio de Salud [MINSA]) and Health Social Security (Seguro Social de Salud [EsSALUD]), while other stakeholders, such as professional medical and psychological associations, municipalities, nongovernmental organizations, and other agencies temporarily provided free telehealth services.^14,15^ However, to our knowledge, no strategies targeted occupational health in various sectors to monitor emerging psychosocial risks during the pandemic and to identify and intervene in adverse mental health outcomes.

Therefore, this narrative review is an attempt to survey the current landscape of OSHS implementation and telehealth strategies in occupational health in Peru. It not only aims to identify current progress and challenges but also to provide a comprehensive framework for the future development of policies and practices in this area, which could significantly contribute to a stronger Peruvian occupational health system and improved well-being and safety among the workers of this country.

## METHODS

This narrative review analyzed technical documents, government reports, regulations, and international organization publications describing the implementation of OSHS and telehealth processes in occupational health in Peru. A systematic search of the main sources of normative information and implementation reports on occupational health and telehealth was conducted in online databases of international technical entities such as the ILO, the World Health Organization, and government agencies, such as the Ministry of Health, the Ministry of Labor and Employment, and the National Institute of Statistics and Informatics, in addition to the repository of norms of *El Peruano*, a major newspaper. The search strategy was also used in scientific databases, such as Scopus and Google Scholar, for relevant documents published in the last 10 years.

The search strategy considered terms related to the implementation of OSHS and occupational telehealth services. Boolean operators were used for strategy construction: (“Occupational health services” OR “occupational health service” OR “occupational medicine services” OR “occupational telehealth” OR “working conditions” OR “employment conditions”) AND (“Telemedicine” OR “eHealth”). Databases that do not allow the use of complex search strategies were searched using each of the above-mentioned terms individually.

Study selection consisted of two phases: first, the titles and abstracts of technical documents or the scope and purpose of the regulations identified in the search were reviewed; the second phase consisted of the full-text review of studies and government documents. Inclusion was based on documents’ relevance to the two topics addressed in this study: OSHS and occupational telehealth. Two researchers independently conducted the bibliography selection process, and any conflicts regarding inclusion were resolved by the principal investigator. A narrative synthesis of the technical and government documents described how they dealt with the study topics.

## RESULTS

This review identified technical documents, government reports, regulations, and publications from international organizations related to the implementation of OSHS and occupational telemedicine processes in the Peruvian context. The systematic search included online databases from government institutions and scientific databases such as Scopus and Google Scholar, using a specifically designed search strategy. A total of 21 relevant documents were identified.

## DISCUSSION

### IMPLEMENTATION OF OCCUPATIONAL HEALTH AND SAFETY SERVICES

Law No. 29783, the Occupational Health and Safety Law, establishes a broad scope for the implementation of occupational health guidelines, covering all economic sectors and services within Peru. This includes employers and workers in both the private and public sectors, the armed forces, the National Police, and self-employed workers. Additionally, Article 36 of this Law establishes the obligation for all employers to provide OSHS, either individually or jointly with other employers, to prevent occupational risks.^7^

The situation of self-employed workers stands out, for which an explanatory definition is provided. The ILO analyzes the concept of self-employment from a global perspective, considering practices in Europe, Asia, Australia, and Mexico. It is understood as economic activity that is dependent on or independent from another organization and may or may not involve a commercial contract, i.e., work conditions similar to those of a regular employee but without a formal employment relationship.^16^ In Peru, it is defined as individuals who work alone or in association to operate a business, enterprise, or profession without other workers in their employ.^17^ Cases of economic and functional dependency on another company fall under the definition of “Contractor” regulated by Law No. 29783, thus including such self-employed workers in preventive activities and OSHS coverage.^18^

Through CENSOPAS, the Peruvian National Institute of Health conducted a cross-sectional study with representative probabilistic sampling to examine working conditions, safety, and health in the country s economically active urban population.^19^ The majority of respondents were men aged 30 to 59 years who worked more than 48 hours per week, mainly from Monday to Saturday. A significant prevalence of occupational risk exposure was found, such as falls, excessive noise, solar radiation, uncomfortable postures, and repetitive movements. Regarding preventive resources and activities, 7.7% of the workers reported being poorly informed about occupational risks. Regarding the identification and evaluation of occupational risks, 35.9% of dependent workers had not had a risk assessment in the last 12 months; 39.4% did not have a prevention delegate/supervisor or a health and safety/hygiene committee at work; 39.3% had not undergone an occupational medical examination in the last 24 months, while 36.5% reported that their workplace did not hold regular meetings to address health and safety issues.^19^

Regarding OSHS provision, 40.7% of the respondents did not have an occupational health service or area in their workplace, which could explain the non-compliance with the above mentioned preventive activities.^19^ The lack of OSHS in Peru contrasts sharply with other contexts, such as the European Union, where coverage ranges up to 100%.^5^ Additionally, the proportion of OSHS coverage in Peru does not include workers in informal or temporary employment, which accounts for nearly 70% of the economically active population.^19^ This falls within the ILO-reported range of informal employment in Latin America (60%-70%).^20^ Furthermore, the ILO reported that, as of 2021, 81,692 companies in Peru had implemented OSHS, a number that has progressively increased over the last 5 years.^21^ This aligns with recent data from the Argentinian National Surveys on Employment, Work, Health, and Safety Conditions, which reported that 48.9% of the working population is covered by OSHS, defined as a service providing occupational medical care and/or surveillance of occupational hygiene and safety measures. However, surveys from Ecuador report that the demand for occupational health professionals is growing beyond its ability to supply trained professionals. In other contexts, there is no academic specialization in occupational health for the various professions involved in OSHS.^20,22^

Given the above, it is necessary to understand OSHS parameters in the workplace. As stipulated in ILO Convention 161 (“Occupational Health Services Convention”),^23^ OSHS are essentially preventive in function. Their main objective is to advise employers, workers, and company representatives on various fundamental areas. This includes promoting and maintaining a safe and healthy work environment that contributes to the physical and mental well-being of workers in relation to their tasks. OSHS are also responsible for ensuring that work tasks are adapted to worker capacities, considering their physical and mental health status. OSHS can be organized in various ways, either as specific services for a single workplace or as shared services. Their composition must be multidisciplinary, adapting to the needs and particular characteristics of the involved work tasks. Additionally, it is crucial for these services to operate in collaboration with other departments of the company, as well as with external agencies, such as public health services, social security institutions, and other entities authorized by the competent authority, thus ensuring adequate coordination and cooperation for effective functioning.^23^

Furthermore, ILO Recommendation 112 on Occupational Health Services states that these services can be organized at the workplace or nearby locations. It is emphasized that the direction of these services should be under a medical professional, who, depending on the circumstances, should be directly responsible for OSHS to the company’s management or the supervisory entity.^24^ However, as mentioned previously, there is a gap in the implementation of OSHS, as well as in the multidisciplinarity of the professionals who provide it. For example, in Europe only Spain, the United Kingdom, and the Nordic countries meet these criteria.^25^

**Figure 1:**
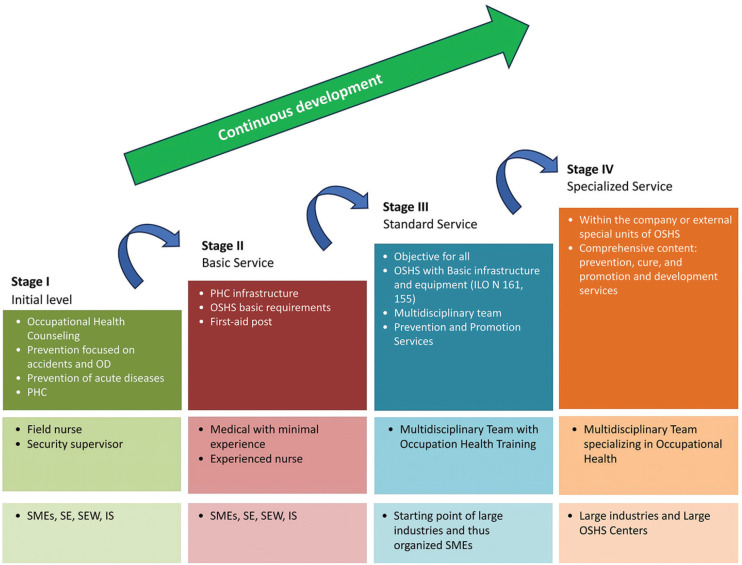
Gradual development of occupational health services.

The World Health Organization, the ILO, and the International Commission on Occupational Health joined efforts to launch the Basic Occupational Health Services initiative in an effort to provide occupational health care to all workers worldwide. The fundamental objectives of these services include protecting the health of workers in their workplaces, promoting their well-being and work capacity, and preventing diseases and accidents.^25^ The initiative proposes a four-stage conceptual model for Basic Occupational Health Services (Figure 1), ranging from an initial level to specialized services, adapting to the increasingly complex needs of occupational health providers. The progress and sophistication of these OSHS are particularly evident in large multinational organizations, since they entail the ability to employ specialized occupational health professionals according to the organization’s specific risks.^25^ In Peru, the lack of a Risk Classification of Economic Activities is evident, although the presence of two groups of economic activities is observed. These groups are distinguished by their inclusion or exclusion in Annex 5 of the Supplementary Risk Work Insurance, according to Supreme Decree No. 008-2022-SA.^26^

ILO = International Labour Organization normative; IS = informal sector; OD = occupational diseases; OSHS = Occupational Health and Safety Services; PHC = primary health care; SE = small enterprise; SEW = self-employed worker; SMEs = small and medium enterprises

However, the latest revision of ISO 45001, which succeeded OHSAS 18001, shows a trend towards less epidemiological surveillance of worker health compared to previous occupational health and safety regulations. It suggests greater attention to occupational safety at the expense of aspects more closely related to occupational health, limiting the actions of OSHS. Prevention and health promotion measures have become their main activity, limited to risk identification and corrective measures. Therefore, it is evident that enforcement of ISO standards is on an observational rather than a mandatory basis in each country.^27^

Approximately 40% of public and private workplaces in Peru have not implemented an OSHS, which indicates not only regulatory non-compliance and worker vulnerability but the employers’ limited ability to carry out occupational health and prevention initiatives.^19^ This problem, which is also evident in other contexts,^20^ involves barriers to and facilitators of OSHS implementation; the success of implementation depends on the commitment of management, effective communication about occupational safety and health with employees, worker participation, efficient resource allocation, the dissemination of positive results (such as the reduction of accidents and occupational diseases and improved worker quality of life), internal incentives, audits, and external incentives.^27,28^ Considering these factors in the OSHS implementation process will promote the perception of a safe and healthy workplace, identify the types of training occupational health professionals need, and lead to greater worker adherence to OSHS recommendations.^27,28^

### TELEHEALTH AND ITS IMPLEMENTATION IN OCCUPATIONAL HEALTH AND SAFETY SERVICES

The progress of telehealth in Peru has been characterized by delayed development of regulatory frameworks that promote the effective utilization of information and communication technologies in the health care sector. A first milestone in this process was Ministerial Resolution No. 297-2012-MINSA, which established a conceptual framework for the incorporation of information and communication technologies in the health sector, defining essential concepts such as telehealth, telemedicine, and digital infrastructure.^29^ Subsequently, Law No. 30421, known as the Telehealth Framework Law and its regulations, provided a comprehensive legal framework delimiting the concepts, guidelines, and general principles needed to implement telehealth in the country.

This law delineated the scope of telehealth, as well as the roles and responsibilities of the various agents involved in its provision, defining four types of telehealth services. First, telemedicine is the provision of medical services at a distance using information and communication technologies, which allows medical consultations, diagnosis, treatment, and patient follow-up without the need for direct physical interaction. Second, telemanagement refers to the remote management of health services, including resource administration, coordination of medical appointments, and management of medical records using digital platforms. Third, teletraining involves the training and education of health care personnel through electronic means, facilitating continuous learning and professional development without the need to commute. Fourth, tele-information, education, and communication initiatives utilize information and communication technologies to disseminate information to the general public on health, healthy lifestyles, health care, and familyand community-related topics.^14,30^

Additionally, Ministerial Resolution No. 1010-2020-MINSA, which approved the “National Telehealth Plan of Peru 2020-2023” technical document, established objectives, goals, and strategic actions for the development and implementation of telehealth based on Telehealth Framework Law guidelines, which to date have not had the desired impact. However, the measurement units for the activities of this plan are imprecise, as they are limited to the development of reports, rather than establishing quantitative indicators that allow monitoring of telehealth services coverage and the identification of gaps in care or the further development of telemanagement.^31^

These normative instruments, as well as their amendments, guidelines, management documents, and regional and local regulation derived from Law No. 30421, have laid the foundations for solid and sustainable telehealth development in Peru. This generates the expectation of expanded access to medical care, a more efficient health care system, and lower health care costs, which proved very useful during the COVID-19 pandemic.^14,32^ However, significant challenges persist, such as the need to train health care personnel in information and communication technologies and to ensure data protection and information security in the context of telehealth, in addition to adapting health services to these innovations and overcoming the related financial difficulties and lack of Internet access.^13,14,32^

No specific telehealth regulations applicable to the field of occupational health or to strengthening OSHS were identified. However, some tele-information, education, and communication initiatives were developed by agencies such as Ministry of Health’s Infosalud and CENSOPAS-INS who, during the pandemic, disseminated information on COVID-19 prevention measures focused on occupational health, specifically targeting delivery workers, caregivers of older adults, market workers, and restaurant workers. These agencies also distributed information on topics related to the management of workplace COVID-19 personal protective equipment waste and the implementation of COVID-19 prevention measures at work.^33,34^ Teletraining was also performed to strengthen COVID-19 prevention and care measures among health care workers.^35^ Finally, “Guidelines for the surveillance of worker health at risk of COVID-19 exposure” and its amendments, prepared by CENSOPAS-INS, was the only technical document to include telehealth-compatible activities that could be performed by OSHS or the health care professionals who assumed occupational health management. The stipulated activities included remote symptom assessment prior to returning or reintegrating workers into on-site work, collective health surveillance of workers, training in COVID-19 prevention and control for OSHS personnel and workers, and remote monitoring of workers with positive cases who were referred to the health insurance system until they were determined fit to return to work.^9^

This study has inherent limitations due to the limited information available on the implementation of OSHS and occupational telehealth in Peru, in addition to the low level of monitoring by the responsible government agencies. These limitations are reflected in the limited availability of data to determine the actual proportion of businesses, both public and private, that have implemented OSHS, as well as the lack of information on the telehealth strategies used in occupational health. However, we have conducted a thorough analysis of all government repositories related to our objectives to provide a synthesis of the current state of the art and the normative frameworks that drive the implementation of OSHS, facilitating the further development of occupational telehealth in Peru. Our results also highlight the need for effective OSHS implementation processes and the dissemination of good practices, guidelines, and technical requirements in occupational telehealth.

## CONCLUSIONS

Approximately 40% of Peruvian businesses have not implemented an OSHS, which is similar to the Latin American and European contexts, apart from any consideration of the informally employed workforce. Some barriers to achieving OSHS implementation are related to employer commitment, the lack of resource allocation, and the lack of regulatory compliance monitoring by oversight agencies, which, in Peru, corresponds to the National Superintendence of Labor Inspection for the private sector, and the National Authority of Civil Service for the public sector. No explicit normative framework for occupational telehealth regulates and guides the actions of OSHS, although telehealth strategies were employed during the COVID-19 pandemic that reached both the working population and OSHS professionals. Telehealth is emerging as a promising tool to strengthen OSHS, and considering it in future normative proposals can ensure its interoperability with health care systems and can lay the groundwork for a national worker health observatory, enabling early intervention in occupational health on sectoral or regional levels.
